# Cerebellar Dysfunction in a Patient with HIV

**DOI:** 10.1155/2014/180743

**Published:** 2014-06-30

**Authors:** Fernando Gonzalez-Ibarra, Waheed Abdul, Sahar Eivaz-Mohammadi, Christopher Foscue, Srinivas Gongireddy, Amer Syed

**Affiliations:** ^1^Department of Internal Medicine, Jersey City Medical Center, Mount Sinai School of Medicine, 355 Grand Street, Jersey City, NJ 07302, USA; ^2^Department of Internal Medicine, Jersey City Medical Center, St. George's University School of Medicine, NJ 07302, USA; ^3^Laureate National Institute of Medicine, Program Director Internal Medicine, Jersey City Medical Center, 355 Grand Street, Jersey City, NJ 07302, USA

## Abstract

A 50-year-old AIDS patient with a CD4 T-cell count of 114/mm^3^ was admitted with cerebellar symptoms of left CN XI weakness, wide-based gait with left-sided dysmetria, abnormal heel-knee-shin test, and dysdiadochokinesia. MRI showed region of hyperintensity in the left inferior cerebellar hemisphere involving the cortex and underlying white matter. Serological tests for HSV1, HSV2, and syphilis were negative. Her CSF contained high protein content and a WBC of 71/mm^3^, predominantly lymphocytes. The CSF was also negative for cryptococcal antigen and VDRL. CSF culture did not grow microbes. CSF PCR assay was negative for HSV1 and HSV2 but was positive for JC virus (1,276 copies). The most likely diagnosis is granule cell neuronopathy (GCN), which can only be definitively confirmed with biopsy and immunohistochemistry.

## 1. Introduction

The most common manifestation of JC virus in HIV infected patients is progressive multifocal leukoencephalopathy (PML) but recent case reports and studies have shown another manifestation called granule cell neuronopathy [1–4]. In this debilitating condition, JC virus infects the granule cells of the cerebellum causing cerebellum degeneration [4]. We report a case featuring a HIV patient with possible granule cell neuronopathy showing the classical symptoms of cerebellar dysfunction.

## 2. Case Report

A 50-year-old Hispanic female presented with a three-day history of bilateral lower extremity and left-upper extremity weakness. She had fallen once at home and was no longer ambulatory due to weakness by the time of admission. The patient had bronchial asthma and had multiple hospitalizations due to exacerbations in the past. She was diagnosed with HIV in 1993 and had been noncompliant with HIV treatment previously. Most recently, she had several months of compliance on HAART therapy that comprised of elvitegravir, cobicistat, emtricitabine, and tenofovir.

At the time of this admission, the patient was alert, awake, oriented, afebrile, and hemodynamically stable. She appeared dehydrated, weak, and cachectic. Respiratory and cardiovascular examinations were within normal limits. Despite complaints of weakness, neurological examination showed 5/5 strength in the upper and lower extremities bilaterally. She had normal deep tendon reflexes, flexor-plantar reflexes, and no sensory deficits. She had a wide-based gait, abnormal dysmetria, abnormal heel-knee-shin test, and dysdiadochokinesia on the left side. She was unable to maintain her balance when rising. Examination of cranial nerves did not show abnormalities except slight weakness of CN XI on the left side, revealed by testing the trapezius and the sternocleidomastoid muscles. The patient was not able to fully raise her left shoulder or turn her head to the left side against resistance.

**Figure 1:**
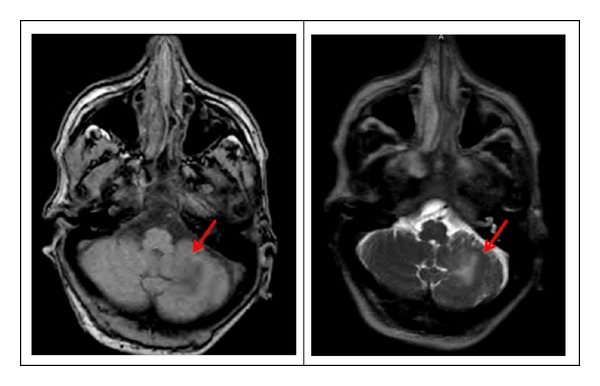
MRI of the brain revealing a region of hyperintense T2 signal in the left inferior cerebellar hemisphere involving the cortex and underlying white matter (shown by arrows). The area of involvement measures 2 cm approximately. No abnormal enhancement was shown after the administration of gadolinium.

Her CD4 T-cell and viral load, drawn the previous month, were 114/mm^3^ and 194,000, respectively. Complete blood count and serum chemistry were within normal range. The patient's ALT, AST, and alkaline phosphatase levels were elevated twofold, most likely due to HAART. During this admission, CT scan of the head showed an area of focal low attenuation in the left cerebellum. T2 weighted MRI showed a 2 cm region of hyperintensity in the left inferior cerebellar hemisphere involving the cortex and underlying white matter (Figure 1).

Serological tests for HSV1, HSV2, and syphilis were negative. A CSF obtained via spinal tap was clear in appearance and contained glucose: 45 mg/dL, protein: 92 mg/dL, RBC: 2/mm^3^, and WBC 71/mm^3^ (lymphocytes: 97, polys: 0, monocytes: 3, eosinophils: 0). The CSF was also VDRL negative and negative for cryptococcal antigen. The culture revealed no growth. The CSF PCR assay was negative for HSV1 and HSV2 but positive for JC virus (1,276 copies).

The clinical impression was for JC-virus CNS infection and granule cell neuronopathy. The patient was discharged on continued HAART therapy with a referral to outpatient physical rehabilitation. Few months later, the patient succumbed to her illness and passed away.

## 3. Discussion

The JC virus is a small, circular, double-stranded-DNA polyomavirus. It is usually acquired in childhood or adolescence, and approximately 70 to 90 percent of the human population carries the virus [4–6]. The virus remains dormant, mostly in the kidneys and lymphoid organs of the host, but may reactivate, spread to the brain, and induce neuronolysis in immunosuppressed patients [1, 4]. JC virus reactivation occurs most frequently in patients with HIV/AIDS but also in transplant patients on immunosuppressive therapy, patients on chemotherapy, and multiple sclerosis patients undergoing natalizumab treatment [1].

Clinical manifestations of the JC virus include classic progressive multifocal leukoencephalopathy (PML), progressive multifocal leukoencephalopathy-immune reconstitution inflammatory syndrome (PML-IRIS), JC virus encephalopathy, JC virus meningitis, and the newly discovered JC virus granule cell neuronopathy [1]. Granule cell neuronopathy is sometimes associated with PML but is a distinct clinical entity [1, 4].

In granule cell neuronopathy, the JC virus induces grey-matter degeneration but does not affect purkinje-cell fibers [1, 2, 4, 7]. The exact nature of the mechanism of Purkinje cell sparing is poorly understood [2, 4]. Recent research has shown that granule cell neuronopathy is a chronic, lytic infection of granule cell neurons in the cerebellum where the diseased granule cells present with hypochromatic, enlarged nuclei [2, 4]. It can occur in isolation or concomitantly with classic PML [4]. The major symptoms are cerebellar signs including ataxic gate, dysdiadochokinesia, abnormal finger to nose test, and heel-knee-shin test [2, 3]. MRI typically shows atrophy of the cerebellum and lesions within the cerebellum. Immunohistochemistry must be performed on brain-biopsy tissue, which was unavailable in this case, to make a definitive diagnosis of this condition [2–4].

In this patient, the possibility of granule cell neuronopathy is most likely but the role of immune reconstitution inflammatory syndrome (IRIS) cannot be ruled without a cerebellum biopsy and subsequent immunohistochemistry workup to show granule cell degeneration. As mentioned before, the patient was recently restarted on HAART therapy. IRIS is demonstrated when the CD4 T-cell counts increase and immunity is recently restored; a paradoxical worsening of the condition is seen with inflammatory reaction in brain lesions [1, 3]. The inflammatory reaction can be demonstrated by contrast enhancement on MRI, which is also seen in our patient's MRI [1].

A compilation of previous case reports found in the literature on JC virus granule cell neuronopathy including demographics, clinical findings, treatments, and the overall outcome is shown in Table 1. The median survival of patients with granule cell neuronopathy is approximately 3 months without HAART therapy and up to 1.8 years in patients on HAART therapy or possibly more depending on CD4 response. HAART therapy increases the T-cell counts in modest amount and is a reliable measure in controlling this disease [2, 3].

## Figures and Tables

**Table 1:** Shows the demographic and clinical findings, treatment, and outcome of JC virus granule cell neuronopathy reported in the literature.

Author	Age	Sex	Primary condition	Location of lesion	Treatment	Outcome
Hecht et al. [8]	15	M	Hyper IgM Type I	Cerebellar hemisphere and vermis	Cidofovir	Nonambulatory
Wüthrich et al. [9]	74	M	Non-small cell lung cancer on chemotherapy	Cerebellar hemisphere	Prednisone	Deceased 4.5 mos after diagnosis
Granot et al. [4]	49	F	Sarcoidosis	Left cerebellar hemisphere	HAART therapy	Deceased few weeks after diagnosis
Tan and Brew [3]	42	M	HIV	Cerebellar hemisphere	HAART therapy and Mefloquine	Deceased 2 years after diagnosis
Koralnik et al. [2]	43	F	HIV	Cerebellar hemisphere	HAART therapy	Deceased 13 years after diagnosis
Dang et al. [7]	25	F	HIV	Right cerebellar hemisphere	HAART therapy	Deceased 4 mos after diagnosis
Dang et al. [7]	34	F	HIV	Cerebellar hemisphere	HAART therapy	Lost in f/u 18 mos after diagnosis
Shang et al. [10]	58	F	HIV and hyper-IgE syndrome	Bilateral middle cerebellar peduncles	HAART therapy	Unchanged in examination after 10 wks
